# Comparative evaluation of transmediastinal and minimally invasive McKeown esophagectomy for esophageal cancer: perioperative and oncologic outcomes

**DOI:** 10.3389/fonc.2025.1644505

**Published:** 2025-08-18

**Authors:** Zhichao Ni, Zigui Zhu, Xin Shi, Xi Xia, Yan Liu, YeHua Cui, Yi Zhang, Jianxin Zhang

**Affiliations:** ^1^ Department of General Thoracic and Cardiovascular Surgery, The Affiliated Nanhua Hospital, Hengyang Medical College, University of South China, Hengyang, Hunan, China; ^2^ Intensive Care Unit, The Affiliated Nanhua Hospital, Hengyang Medical College, University of South China, Hengyang, Hunan, China; ^3^ Department of General Surgery, Xupu County Traditional Chinese Medicine Hospital, Huaihua, Hunan, China; ^4^ Department of Thoracic, Cardiac, and Breast Surgery, Changsha Fourth Hospital, Changsha, Hunan, China

**Keywords:** esophageal cancer, transmediastinal esophagectomy, McKeown procedure, minimally invasive surgery, surgical outcomes, postoperative complications

## Abstract

**Background:**

Esophageal squamous cell carcinoma remains a major health burden in China, where surgical resection is the mainstay of curative therapy. The conventional minimally invasive McKeown esophagectomy (MIE-McKeown), although oncologically effective, entails transthoracic access and single-lung ventilation, often resulting in higher postoperative morbidity. Thoraco-laparoscopic transmediastinal esophagectomy has emerged as a novel alternative that may mitigate these risks while preserving oncologic integrity.

**Methods:**

A retrospective cohort study was conducted involving 268 patients with resectable middle or lower thoracic esophageal squamous cell carcinoma (clinical stage I–III), including 131 who underwent transmediastinal esophagectomy and 137 who received MIE-McKeown. Outcomes assessed included operative time, intraoperative blood loss, lymph node yield, complication profiles, recovery indicators, quality of life (EORTC QLQ-C30), and 6-month disease-free survival (DFS). Statistical comparisons were performed using t-tests, χ² tests, and multivariate logistic regression.

**Results:**

The transmediastinal esophagectomy group exhibited significantly shorter operative time (197.2 ± 25.9 *vs.* 286.5 ± 32.1 min, *P*<0.001) and reduced blood loss (155.4 ± 40.2 *vs.* 260.7 ± 65.1 mL, *P*<0.001). Time to oral intake (4.6 ± 1.1 *vs.* 6.2 ± 1.3 days, *P*<0.001), drainage duration (3.8 ± 0.5 *vs.* 4.4 ± 0.7 days, *P*<0.001), and hospital stay (9.3 ± 1.8 *vs.* 11.1 ± 2.2 days, *P*<0.001) were all significantly improved in the transmediastinal esophagectomy group. The incidence of Clavien–Dindo grade ≥III complications was lower (7.6% *vs.* 16.0%, *P*=0.043), particularly pneumonia (7.6% *vs.* 18.2%, *P*=0.009) and recurrent laryngeal nerve injury (4.6% *vs.* 11.7%, *P*=0.031). Lymph node harvest was comparable (21.4 ± 6.2 *vs.* 22.1 ± 5.9, *P*=0.344). Three-month quality-of-life scores were higher in the transmediastinal esophagectomy group for global health (73.4 ± 12.1 *vs.* 66.5 ± 13.4, *P*=0.005), physical functioning (78.2 ± 11.8 *vs.* 70.6 ± 13.6, *P*=0.008), and role functioning (72.1 ± 14.2 *vs.* 64.3 ± 15.1, *P*=0.011). The 6-month DFS rates were similar between groups (93.1% *vs.* 91.2%, log-rank *P*=0.327).

**Conclusions:**

Thoraco-laparoscopic transmediastinal esophagectomy is a safe, effective, and minimally invasive alternative to the McKeown approach in selected esophageal squamous cell carcinoma patients. It provides superior perioperative outcomes and enhanced recovery without compromising short-term oncologic efficacy.

## Introduction

Esophageal cancer ranks among the most lethal malignancies globally, posing a substantial burden to public health systems, particularly in high-incidence regions such as China. According to GLOBOCAN 2020 statistics, esophageal cancer accounted for approximately 604,000 new cases and 544,000 deaths worldwide, ranking eighth in cancer incidence and sixth in cancer mortality globally (1). China alone contributed more than 50% of both incidence and mortality, with 324,000 new cases and 301,000 deaths, reflecting a striking geographic disparity in disease burden (1, 2). Squamous cell carcinoma constitutes over 90% of esophageal malignancies in East Asia and remains the predominant histologic subtype in China (3). Despite advances in multimodal therapies—including chemotherapy, radiotherapy, and immune checkpoint inhibition—surgical resection continues to serve as the cornerstone of curative-intent treatment for resectable esophageal cancer (4). However, the optimal surgical approach remains debated, especially in balancing oncologic efficacy with perioperative safety and long-term functional outcomes.

Historically, the Sweet procedure—employing a left thoracotomy—was widely practiced in China but gradually fell out of favor due to its limited exposure to the upper mediastinum and suboptimal lymphadenectomy, particularly in the paratracheal and recurrent laryngeal nerve chains (5). This limitation resulted in a high rate of nodal recurrence (up to 40%) in the upper mediastinum and contributed to stagnation of 5-year survival rates at 30–40% over recent decades (6). With the adoption of standardized lymphadenectomy and minimally invasive techniques, the McKeown three-field esophagectomy—utilizing a right thoracoscopic, laparoscopic, and cervical approach—has become the mainstream strategy (7–9). The McKeown approach enables superior exposure of the upper mediastinum and facilitates radical lymph node dissection, particularly around bilateral recurrent laryngeal nerves, improving long-term oncological outcomes (10). Nevertheless, this technique necessitates transthoracic access and single-lung ventilation, both of which contribute to substantial cardiopulmonary burden, higher rates of postoperative pneumonia, recurrent laryngeal nerve palsy, pleural effusion, and prolonged recovery (7, 11).

In response to these challenges, thoraco-laparoscopic transmediastinal esophagectomy has emerged as a less invasive alternative that bypasses transthoracic access while maintaining an adequate oncologic field. This procedure leverages natural anatomical planes via a transcervical route and transhiatal access under thoraco-laparoscopic guidance, obviating the need for one-lung ventilation or thoracic cavity entry (12). Early evidence suggests that transmediastinal esophagectomy may significantly reduce operative time, intraoperative blood loss, and pulmonary complications—thereby enabling enhanced recovery and decreased hospitalization costs (8, 10, 13). Furthermore, the transcervical dissection provides excellent visualization of the left recurrent laryngeal nerve chain, enabling en bloc lymphadenectomy without the spatial limitations imposed by thoracic structures. Our institutional experience and surgical atlas development support the technical feasibility and safety of this approach (14). However, robust comparative evidence assessing its clinical efficacy relative to standard McKeown esophagectomy in real-world practice remains limited, particularly in terms of short-term recovery profiles, complication spectra, and early oncological outcomes. Moreover, while the McKeown approach is associated with improved lymph node access due to the cervical incision, it also allows for easier cervical management of anastomotic complications, particularly anastomotic leaks. Cervical anastomosis, although associated with a slightly higher leak rate compared to intrathoracic anastomosis, is generally considered safer due to the lower risk of mediastinitis and the feasibility of local drainage or conservative treatment. This potential trade-off between anastomotic safety and surgical radicality further underscores the need for comprehensive comparison of these two approaches.

To address this critical knowledge gap, we conducted a retrospective comparative study evaluating thoraco-laparoscopic transmediastinal esophagectomy versus conventional minimally invasive McKeown esophagectomy in patients with resectable middle or lower thoracic esophageal squamous cell carcinoma. Drawing upon a well-matched cohort and comprehensive perioperative metrics, we tested the hypothesis that transmediastinal esophagectomy offers comparable oncologic efficacy while significantly improving perioperative safety and recovery trajectories. We assessed operative parameters, postoperative complication rates, lymph node yield, short-term disease-free survival, and quality of life outcomes using validated metrics. This study provides novel evidence supporting the clinical utility of a non-transthoracic, minimally invasive esophagectomy paradigm and informs surgical decision-making in the era of enhanced recovery after surgery and patient-centered cancer care.

## Methods

### Study design and patients

This retrospective observational study was conducted at The Affiliated Nanhua Hospital, Hengyang Medical College, University of South China, and was approved by the Institutional Review Board of the The Affiliated Nanhua Hospital, Hengyang Medical College, University of South China. The study included consecutive patients with histologically confirmed thoracic esophageal squamous cell carcinoma who underwent curative-intent resection between June 1, 2022, and June 28, 2024. Eligible patients were required to meet the following inclusion criteria: (1) tumor located in the middle or lower thoracic esophagus; (2) clinical stage I–III based on preoperative contrast-enhanced CT, endoscopic ultrasonography, and PET-CT as appropriate; (3) no distant metastases or other primary malignancies; (4) no history of neoadjuvant therapy; and (5) ASA physical status ≤ III. Patients with cervical esophageal cancer, severe cardiopulmonary dysfunction, or history of thoracic surgery were excluded.

A total of 268 patients met the inclusion criteria and were enrolled in the final analysis. Among them, 131 patients underwent thoraco-laparoscopic transmediastinal esophagectomy (transmediastinal group), and 137 received conventional minimally invasive McKeown esophagectomy (McKeown group). Baseline demographic and clinical characteristics were well balanced between the two groups.

### Baseline data collection

Baseline demographic and clinical data were collected prospectively and retrieved from the institutional electronic medical record system. Age, sex, body mass index (BMI), smoking and alcohol history, and comorbidities including hypertension, diabetes mellitus, and chronic obstructive pulmonary disease (COPD) were documented during the preoperative evaluation. The Charlson Comorbidity Index was calculated to quantify baseline comorbidity burden. Laboratory parameters including serum albumin, carcinoembryonic antigen (CEA), squamous cell carcinoma antigen (SCC), and cytokeratin 19 fragment (CYFRA21-1) were measured within one week prior to surgery using standardized chemiluminescence immunoassays in the hospital’s central laboratory. Nutritional risk was assessed using the NRS-2002 scale (15), administered by trained nutritionists. Tumor location and clinical TNM staging were determined through preoperative contrast-enhanced CT, endoscopic ultrasonography, and PET-CT when clinically indicated. Histologic grade was confirmed by pathology following endoscopic biopsy. All variables were defined and recorded prior to surgical allocation to minimize bias and ensure comparability across groups.

### Surgical procedures

All procedures were performed by experienced thoracic surgeons with >10 years of esophagectomy experience, under standardized anesthesia protocols. In the transmediastinal group, surgery was performed in the supine position with the head turned to the right and legs separated. A cervical incision was made along the medial border of the left sternocleidomastoid muscle. After placement of a protective sleeve and CO_2_ insufflation, thoracoscopic instruments were introduced to dissect the upper and middle thoracic esophagus transmediastinally, with special attention to preserving the thoracic duct and azygos vein and clearing the left recurrent laryngeal nerve lymph nodes.

Simultaneously, a laparoscopic team performed dissection of the lower thoracic esophagus, abdominal esophagus, and stomach. Standard steps included mobilization of the greater curvature, ligation of short gastric vessels and left gastric vessels, and lymphadenectomy around the left gastric artery, lesser curvature, and esophagogastric junction. A 4-cm-wide tubular stomach was constructed along the lesser curvature. The gastric conduit was then pulled up through the posterior mediastinal route and anastomosed to the proximal esophageal stump at the neck using a circular stapler.

In the McKeown group, patients received right thoracoscopic esophageal mobilization in the left lateral decubitus position with single-lung ventilation. The azygos vein was divided, and recurrent laryngeal nerve lymphadenectomy was performed bilaterally. Cervical and abdominal phases followed standard McKeown protocol, including tubular stomach creation and cervical esophagogastric anastomosis. Chest tubes were placed in all McKeown patients but omitted in the transmediastinal group unless intraoperative pleural breach occurred.

### Outcome measures

Primary outcomes included intraoperative metrics (operative time, estimated blood loss), lymphadenectomy details (number and stations of dissected lymph nodes), and postoperative recovery indicators (drainage duration, time to first oral intake, length of hospital stay, and hospitalization cost). Postoperative complications were assessed up to 90 days and categorized according to the Clavien–Dindo classification (16, 17). Specific complications of interest included pneumonia, pleural effusion, recurrent laryngeal nerve injury, anastomotic leakage, chylothorax, arrhythmia, pulmonary embolism, and respiratory failure. Major complications were defined as Clavien–Dindo grade ≥ III (16, 17).

Secondary outcomes included functional status and quality of life at 3 months, assessed using the EORTC QLQ-C30 questionnaire (18), and short-term oncologic outcomes including 6-month disease-free survival (DFS), defined as the time from surgery to the first radiologic or histologic evidence of recurrence.

### Statistical analysis

All statistical analyses were performed using SPSS version 24.0 (IBM Corp., Armonk, NY, USA). Continuous variables were reported as mean ± standard deviation and compared using independent-sample *t* tests. Categorical variables were expressed as numbers and percentages and compared using the *χ^2^* test or Fisher’s exact test as appropriate. Univariate and multivariate logistic regression analyses were conducted to identify independent predictors of favorable postoperative outcomes, defined as absence of Clavien–Dindo grade ≥ III complications within 90 days. Odds ratios (ORs) with 95% confidence intervals (CIs) were reported. Kaplan–Meier method was used for survival analysis, and log-rank tests were used for group comparisons. A two-sided *P* value < 0.05 was considered statistically significant.

## Results

### Baseline demographic and clinical characteristics of the two surgical groups

A total of 268 patients were included, comprising 131 patients who underwent thoraco-laparoscopic transmediastinal esophagectomy (transmediastinal group) and 137 who received minimally invasive McKeown esophagectomy (McKeown group). As shown in **Table 1**, baseline characteristics were well balanced between the groups. There were no statistically significant differences in age (62.4 ± 4.9 *vs.* 62.7 ± 5.2 years, *P*=0.628), BMI (24.0 ± 1.9 *vs.* 24.4 ± 2.1 kg/m², *P*=0.104), Charlson Comorbidity Index (2.1 ± 0.4 *vs.* 2.2 ± 0.5, P=0.073), preoperative albumin (39.5 ± 3.0 *vs.* 39.1 ± 3.2 g/L, *P*=0.293), CEA, SCC, CYFRA21–1 levels, and NRS-2002 nutritional risk scores. Similarly, proportions of males (74.8% *vs.* 71.5%, *P*=0.545), smoking history (55.7% *vs.* 56.2%, *P*=0.937), alcohol use, and comorbidities such as hypertension, diabetes, and COPD were comparable (*P*>0.05 for all). No differences were observed in tumor location, clinical TNM staging, or histological grading (*P*>0.05), confirming the groups were well matched for subsequent outcome comparisons.

**Table 1:** Baseline characteristics of the two surgical groups.

Variable	Transmediastinal group (n=131)	McKeown group (n=137)	*t*/*χ^2^*	*P*-value
Age (years)	62.4 ± 4.9	62.7 ± 5.2	-0.486	0.628
BMI (kg/m^2^)	24.0 ± 1.9	24.4 ± 2.1	-1.633	0.104
Charlson Comorbidity Index	2.1 ± 0.4	2.2 ± 0.5	-1.803	0.073
Preoperative serum albumin (g/L)	39.5 ± 3.0	39.1 ± 3.2	1.055	0.293
Preoperative CEA (ng/mL)	3.8 ± 1.5	4.0 ± 1.7	-1.019	0.309
NRS-2002 score	2.6 ± 0.7	2.7 ± 0.6	-1.257	0.210
Serum SCC (ng/mL)	1.9 ± 0.4	2.0 ± 0.5	-1.803	0.073
Serum CYFRA21-1 (ng/mL)	4.3 ± 1.1	4.5 ± 1.3	-1.357	0.176
Male sex	98 (74.8%)	98 (71.5%)	0.366	0.545
Smoking history	73 (55.7%)	77 (56.2%)	0.006	0.937
Alcohol use	57 (43.5%)	60 (43.8%)	0.002	0.963
Hypertension	42 (32.1%)	39 (28.5%)	0.410	0.522
Diabetes	25 (19.1%)	28 (20.4%)	0.077	0.781
COPD	11 (8.4%)	10 (7.3%)	0.112	0.738
Clinical stage			0.153	0.926
Clinical stage I	28 (21.4%)	32 (23.3%)		
Clinical stage II	65 (49.6%)	66 (48.2%)		
Clinical stage III	38 (29.0%)	39 (28.5%)		
Tumor location			0.030	0.863
Tumor location (Middle thoracic)	75 (57.3%)	77 (56.2%)		
Tumor location (Lower thoracic)	56 (42.7%)	60 (43.8%)		
Histological grade			0.249	0.883
Histological grade (Well)	20 (15.3%)	22 (16.1%)		
Histological grade (Moderate)	69 (52.7%)	68 (49.6%)		
Histological grade (Poor)	42 (32.1%)	47 (34.3%)		
Clinical T stage			0.058	0.809
Clinical T stage (T1-T2)	65 (49.6%)	70 (51.1%)		
Clinical T stage (T3)	66 (50.4%)	67 (48.9%)		
Clinical N stage			0.110	0.740
Clinical N stage (N0)	81 (61.8%)	82 (59.9%)		
Clinical N stage (N1-N2)	50 (38.2%)	55 (40.1%)		

Data are presented as mean ± standard deviation or number (percentage). Comparisons were made using independent-samples t-test for continuous variables and chi-square test for categorical variables. CEA, carcinoembryonic antigen; SCC, squamous cell carcinoma antigen; CYFRA21-1, cytokeratin 19 fragment; NRS-2002, Nutritional Risk Screening 2002.

### Intraoperative metrics: operative time, blood loss, and lymphadenectomy outcomes

**Figure 1:**
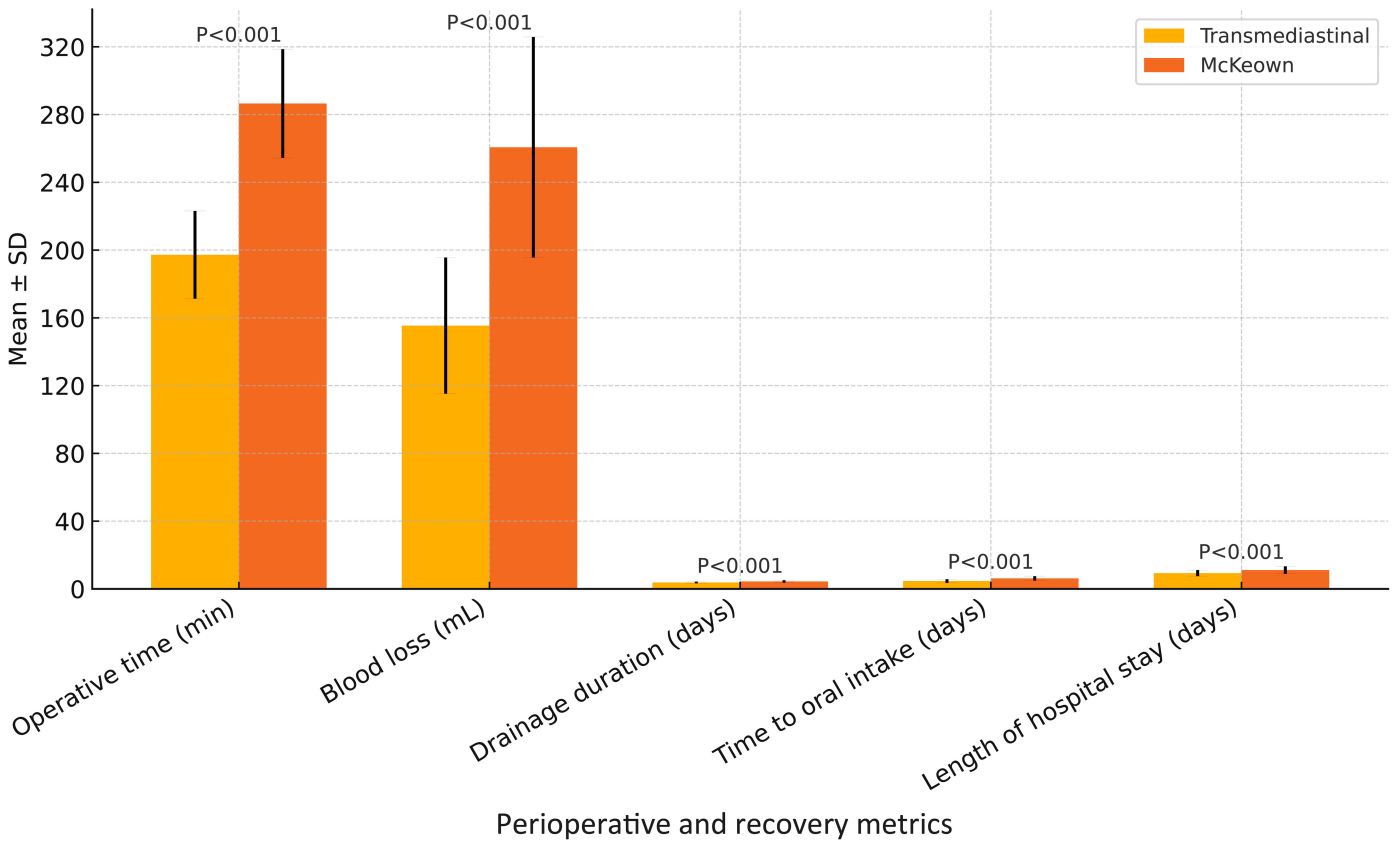
Comparison of perioperative and recovery metrics between transmediastinal and McKeown esophagectomy. Mean values with standard deviations are shown for operative time, intraoperative blood loss, drainage duration, time to oral intake, and length of hospital stay. The transmediastinal group demonstrated significantly shorter operative time and faster postoperative recovery across all parameters (*P* < 0.001 for all comparisons).

As shown in **Table 2**, the transmediastinal group had a significantly shorter operative time (197.2 ± 25.9 *vs.* 286.5 ± 32.1 min, *P*<0.001) and less intraoperative blood loss (155.4 ± 40.2 *vs.* 260.7 ± 65.1 mL, *P*<0.001). Although both groups achieved adequate lymphadenectomy, the number of dissected lymph nodes was comparable (21.4 ± 6.2 *vs.* 22.1 ± 5.9, *P*=0.344). Postoperatively, the transmediastinal group experienced shorter drainage duration (3.8 ± 0.5 *vs.* 4.4 ± 0.7 days, *P*<0.001), earlier time to oral intake (4.6 ± 1.1 *vs.* 6.2 ± 1.3 days, *P*<0.001), and reduced length of hospital stay (9.3 ± 1.8 *vs.* 11.1 ± 2.2 days, *P*<0.001), indicating faster recovery and better resource efficiency. These findings are visualized in **Figure 1**.

**Table 2:** Intraoperative and early recovery metrics in two surgical groups.

Variable	Transmediastinal group (n=131)	McKeown group (n=137)	*t*	*P*-value
Operative time (min)	197.2 ± 25.9	286.5 ± 32.1	-24.997	<0.001
Intraoperative blood loss (mL)	155.4 ± 40.2	260.7 ± 65.1	-15.848	<0.001
Lymph nodes dissected (n)	21.4 ± 6.2	22.1 ± 5.9	-0.947	0.344
Drainage duration (days)	3.8 ± 0.5	4.4 ± 0.7	-8.043	<0.001
Time to oral intake (days)	4.6 ± 1.1	6.2 ± 1.3	-10.853	<0.001
Length of hospital stay (days)	9.3 ± 1.8	11.1 ± 2.2	-7.312	<0.001

Data are expressed as mean ± standard deviation. Comparisons between groups were performed using independent-samples t-test. Statistically significant differences (*P* < 0.05) are observed in operative time, blood loss, drainage duration, and time to oral intake.

### Postoperative complications and morbidity profile based on clavien–dindo classification

As summarized in **Table 3**, the transmediastinal group had a significantly lower overall complication rate compared to the McKeown group (18.3% vs. 32.1%, *P*=0.009). Specifically, the incidence of pneumonia (10 *vs.* 25 cases), recurrent laryngeal nerve injury (6 *vs.* 16 cases), pleural effusion (5 *vs.* 10 cases), and anastomotic leakage (3 vs. 6 cases) was numerically lower in the transmediastinal group. Major complications (Clavien–Dindo grade ≥ III) occurred less frequently in the transmediastinal group (10 *vs.* 22 cases). It is noteworthy that some patients experienced multiple complications. Among the patients who developed anastomotic leakage, none in the transmediastinal group required reoperation; all were managed conservatively with fasting, intravenous antibiotics, and external drainage. In contrast, one patient in the McKeown group underwent surgical re-intervention due to a persistent cervical leak and signs of systemic sepsis. These observations suggest that while leak incidence was low in both groups, the severity and clinical management differed slightly.

**Table 3:** Postoperative complications in two surgical groups.

Complication	Transmediastinal group (n=131)	McKeown group (n=137)	*χ^2^*	*P*-value
Overall complications, n (%)	24 (18.3%)	44 (32.1%)	6.731	0.009
Pneumonia, n	10	2		
Recurrent laryngeal nerve injury, n	6	16		
Pleural effusion, n	5	10		
Anastomotic leakage, n	3	6		
Arrhythmia, n	2	4		
Chylothorax, n	1	2		
Pulmonary embolism, n	0	1		
Respiratory failure, n	0	1		
Clavien–Dindo ≥ III complications, n	10	22		

Data are expressed as number (percentage). Statistical analysis was performed using the chi-square test. Some patients may have experienced multiple complications.

### Three-month functional prognosis, quality of life, and short-term oncological outcomes

**Figure 2:**
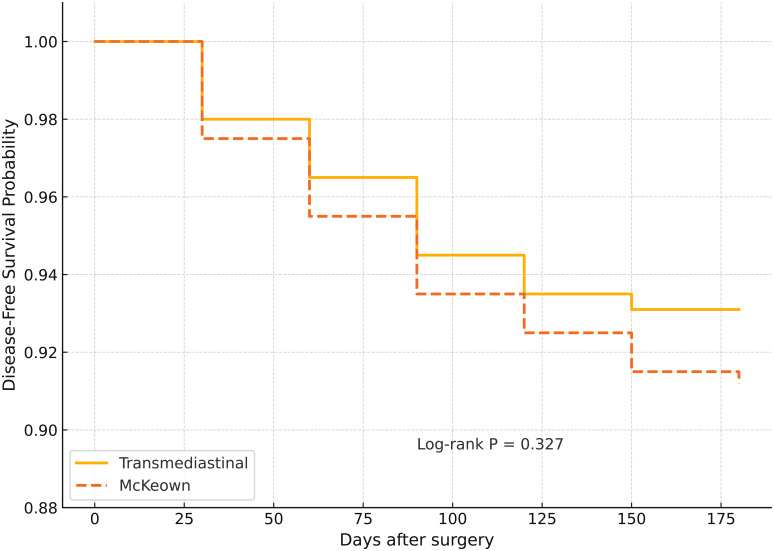
Kaplan–Meier curves for 6-month disease-free survival (DFS) in patients undergoing transmediastinal versus McKeown esophagectomy. Kaplan–Meier estimates showed a slightly higher 6-month disease-free survival rate in the transmediastinal group (93.1%) compared to the McKeown group (91.2%), with no statistically significant difference (log-rank *P* = 0.327). This indicates comparable short-term oncologic efficacy between the two surgical approaches.

**Figure 3:**
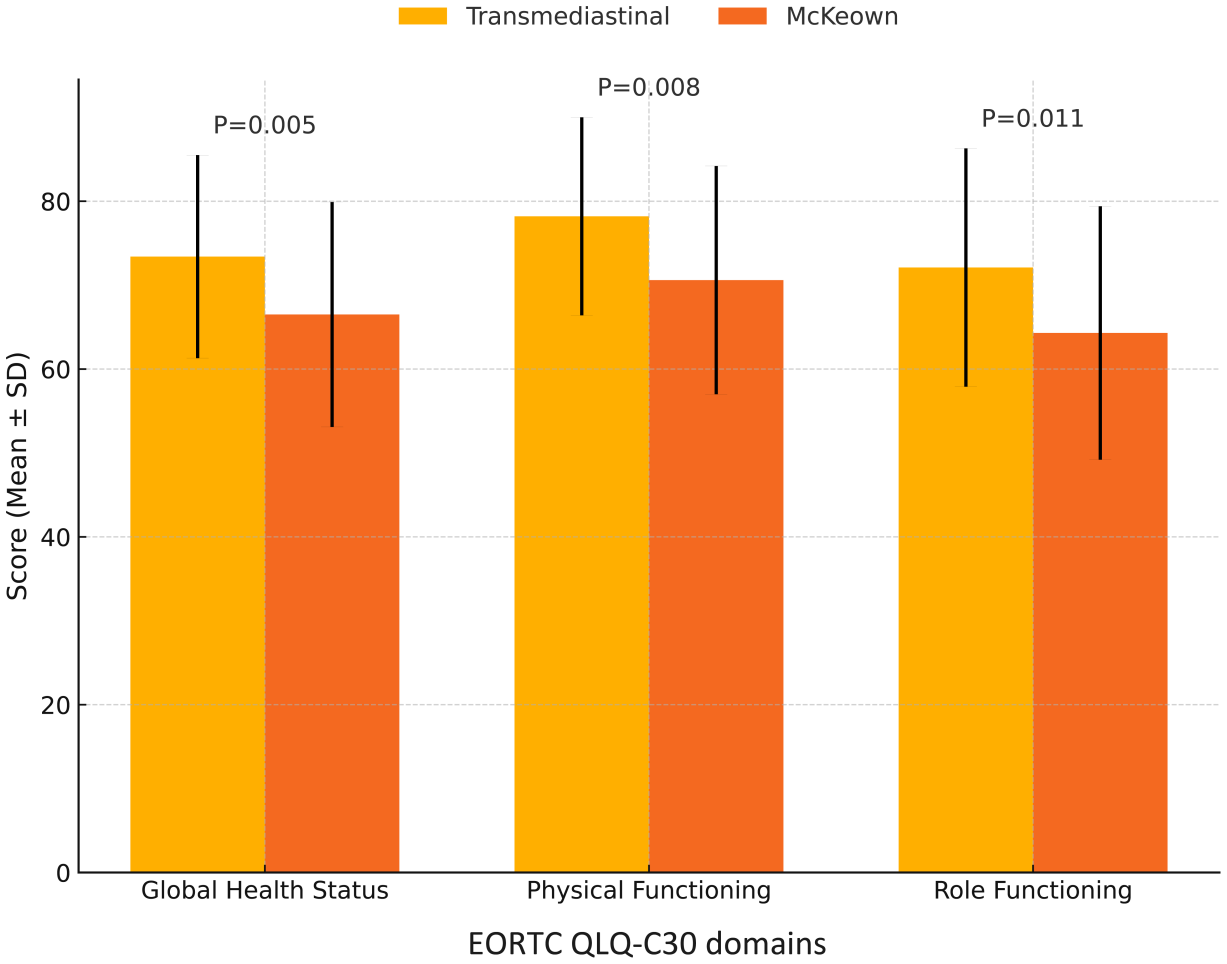
Comparison of EORTC QLQ-C30 quality of life scores at 3 months postoperatively. Patients in the transmediastinal group reported significantly higher scores in global health status (*P* = 0.005), physical functioning (*P* = 0.008), and role functioning (*P* = 0.011) at 3 months after surgery, as measured by the EORTC QLQ-C30 questionnaire. These results suggest a better early postoperative quality of life following transmediastinal esophagectomy.

At 90 days postoperatively, a significantly higher proportion of patients in the transmediastinal group remained free of major complications (Clavien–Dindo grade ≥III: 7.6% *vs.* 16.0%; *P*=0.043). The 6-month disease-free survival (DFS) rate was similar between groups (93.1% *vs.* 91.2%; log-rank *P*=0.327), as shown in **Figure 2**. Quality of life, assessed using the EORTC QLQ-C30 questionnaire, was notably better in the transmediastinal group at 3 months. Scores for global health status (73.4 ± 12.1 *vs.* 66.5 ± 13.4, *P*=0.005), physical functioning (78.2 ± 11.8 *vs.* 70.6 ± 13.6, *P*=0.008), and role functioning (72.1 ± 14.2 *vs.* 64.3 ± 15.1, *P*=0.011) all favored the transmediastinal approach (**Figure 3**).

### Univariate and multivariate logistic regression for predictors of favorable postoperative outcomes

**Figure 4:**
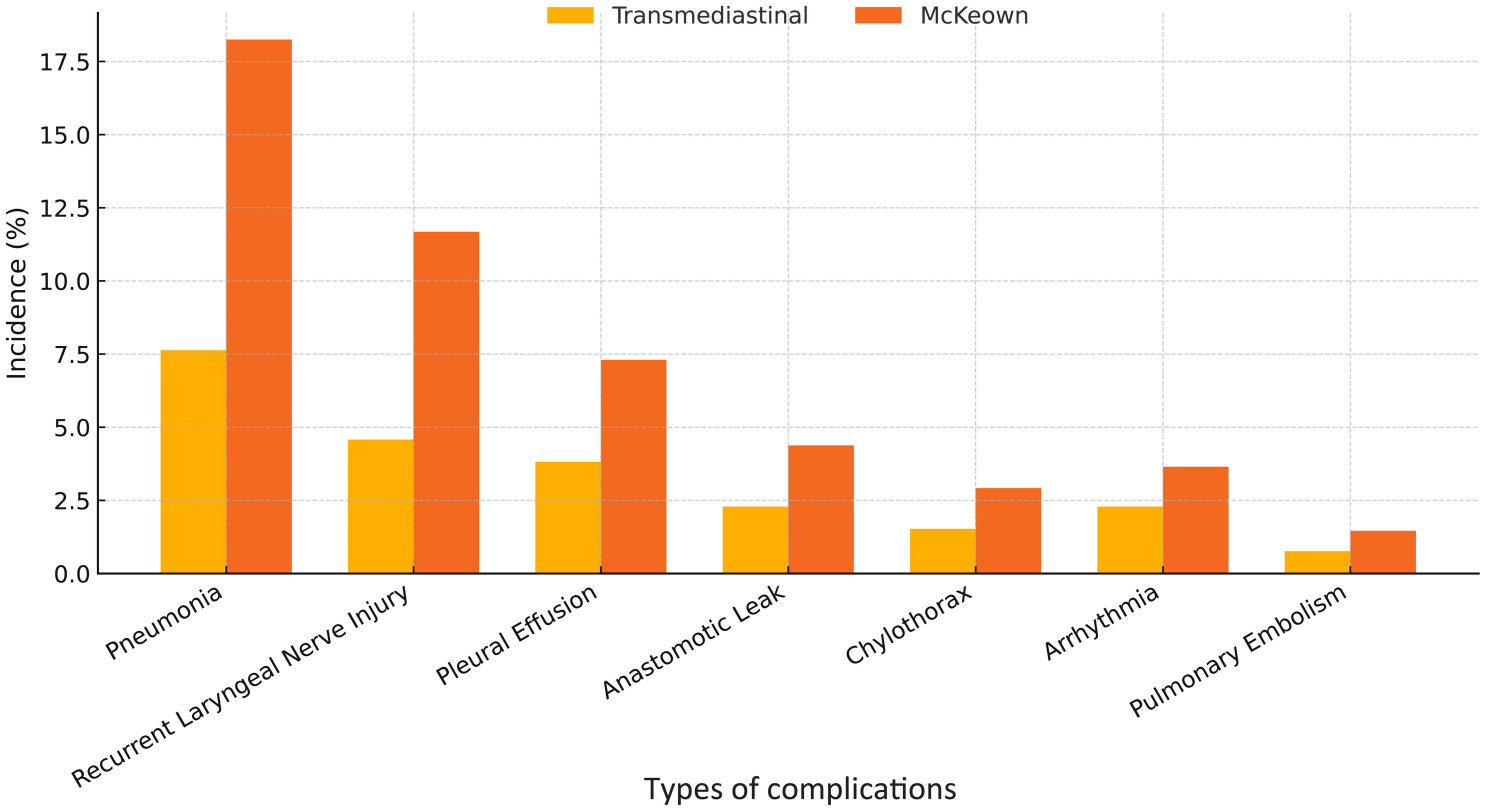
Incidence of major postoperative complications following transmediastinal versus McKeown esophagectomy. The bar chart compares the rates of key postoperative complications including pneumonia, recurrent laryngeal nerve injury, pleural effusion, and anastomotic leakage. The transmediastinal group consistently showed lower incidence across all categories, with an overall complication rate of 18.3% compared to 32.1% in the McKeown group.

Univariate and multivariate logistic regression analyses were conducted to identify predictors of favorable short-term outcomes, defined as the absence of Clavien–Dindo grade ≥ III complications within 90 days (**Table 4** and **Figure 4**).

**Table 4:** Univariate and multivariate logistic regression for predictors of favorable postoperative outcomes.

Variable	b	S.E.	Wald *χ^2^*	Univariate OR (95% CI)	*P*-value (Univariate)	b^a^	S.E.^a^	Wald *χ^2^* ^a^	Multivariate OR (95% CI)^a^	*P*-value (Multivariate)^a^
Surgical approach (Transmediastinal)	0.667	0.268	6.194	1.948 (1.152–3.295)	0.013	0.631	0.281	5.043	1.879 (1.084–3.260)	0.025
Operative time (per min)	-0.012	0.004	9.000	0.988 (0.980–0.996)	0.003	-0.032	0.012	7.111	0.969 (0.946–0.992)	0.008
Blood loss (per mL)	-0.011	0.005	4.840	0.989 (0.979–0.999)	0.028	-0.142	0.216	0.432	0.868 (0.568–1.325)	0.511
Clinical stage (III)	-0.545	0.256	4.532	0.580 (0.351–0.958)	0.033	-0.495	0.243	4.150	0.610 (0.379–0.981)	0.042
Age (per year)	0.012	0.014	0.734	1.012 (0.985–1.040)	0.392					
BMI (per kg/m^2^)	-0.031	0.053	0.342	0.970 (0.874–1.075)	0.559					
Albumin (per g/L)	0.015	0.030	0.250	1.015 (0.957–1.079)	0.617					
CEA (per ng/mL)	-0.042	0.058	0.525	0.959 (0.855–1.078)	0.469					
SCC (per ng/mL)	-0.133	0.166	0.641	0.875 (0.632–1.211)	0.423					
CYFRA21-1 (per ng/mL)	-0.097	0.095	1.043	0.908 (0.751–1.098)	0.307					
NRS-2002 score	-0.212	0.205	1.070	0.809 (0.540–1.212)	0.301					
Male sex (yes)	0.058	0.243	0.057	1.061 (0.662–1.701)	0.811					
Smoking history (yes)	-0.014	0.249	0.003	0.993 (0.612–1.623)	0.958					
Alcohol use (yes)	0.072	0.245	0.086	1.070 (0.652–1.761)	0.770					
Diabetes (yes)	-0.204	0.282	0.523	0.820 (0.470–1.422)	0.470					
Hypertension (yes)	-0.103	0.248	0.173	0.901 (0.562–1.451)	0.678					
COPD (yes)	-0.390	0.415	0.882	0.683 (0.301–1.548)	0.348					
Charlson comorbidity index	-0.116	0.164	0.501	0.887 (0.652–1.232)	0.479					
Tumor location (Middle thoracic)	0.054	0.249	0.047	1.058 (0.646–1.727)	0.828					
Histological grade (Well)	-0.017	0.288	0.004	0.982 (0.558–1.732)	0.950					
Clinical T stage (T1–T2)	-0.133	0.276	0.232	0.876 (0.512–1.528)	0.630					
Clinical N stage (N0)	-0.171	0.268	0.408	0.839 (0.501–1.422)	0.523					

a indicates variables with *P* < 0.05 in univariate analysis that were included in multivariate logistic regression. OR, odds ratio; CI, confidence interval; S.E, standard error.

In univariate analysis, the transmediastinal approach (OR=1.948, 95% CI: 1.152–3.295, *P*=0.013), shorter operative time (OR=0.988 per min, 95% CI: 0.980–0.996, *P*=0.003), and lower intraoperative blood loss (OR=0.989 per mL, 95% CI: 0.979–0.999, *P*=0.028) were significantly associated with favorable outcomes. Conversely, clinical stage III was associated with increased complication risk (OR=0.580, 95% CI: 0.351–0.958, P=0.033).

In multivariate analysis, the transmediastinal surgical approach remained an independent protective factor (adjusted OR=1.879, 95% CI: 1.084–3.260, *P*=0.025), along with shorter operative time (adjusted OR=0.969 per min, 95% CI: 0.946–0.992, *P*=0.008). Clinical stage III continued to predict worse outcomes (adjusted OR=0.610, 95% CI: 0.379–0.981, *P*=0.042). Intraoperative blood loss was not independently significant in the multivariate model (*P*=0.511).

## Discussion

**Figure 5:**
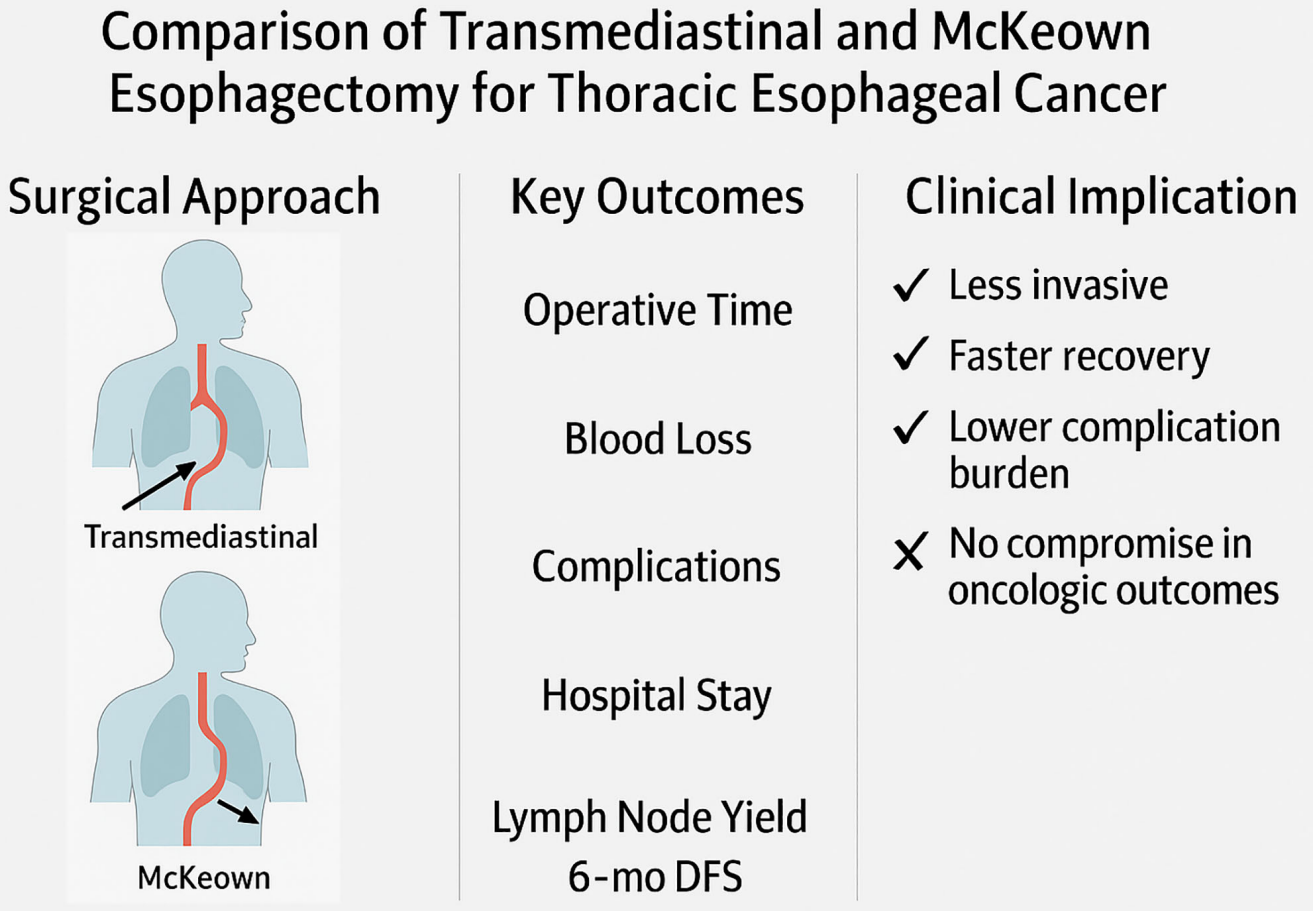
Comparative overview of surgical strategy, perioperative outcomes, and clinical impact between transmediastinal and McKeown esophagectomy.

This study presents a comprehensive comparison between thoraco-laparoscopic transmediastinal esophagectomy and the conventional minimally invasive McKeown procedure for the treatment of thoracic esophageal squamous cell carcinoma. By analyzing perioperative metrics, postoperative complication profiles, and short-term oncological outcomes in a well-matched cohort, we demonstrate that transmediastinal esophagectomy provides significant clinical advantages. Specifically, transmediastinal esophagectomy was associated with reduced operative time, less intraoperative blood loss, lower rates of pneumonia and recurrent laryngeal nerve injury, and accelerated postoperative recovery, while maintaining comparable lymph node dissection yields and 6-month DFS. These findings substantiate our initial hypothesis that a transmediastinal approach can serve as a less invasive yet equally effective alternative to the traditional transthoracic route in selected patients with thoracic esophageal squamous cell carcinoma (**Figure 5**).

The novelty and clinical relevance of transmediastinal esophagectomy lie in its ability to eliminate the need for thoracotomy or single-lung ventilation, two key contributors to cardiopulmonary stress and postoperative morbidity in esophageal surgery (19). The McKeown procedure, though widely recognized for its radicality, requires transthoracic access which entails one-lung ventilation, rib spreading, and mediastinal manipulation—all of which may impair pulmonary function and increase perioperative risk (20, 21). Transmediastinal esophagectomy, in contrast, utilizes natural anatomical planes accessed via the cervical and transhiatal routes, guided by thoraco-laparoscopy and CO_2_ insufflation (22). This strategy offers a stable operative field with direct visualization of the upper mediastinum, especially the left recurrent laryngeal nerve and tracheoesophageal groove, enabling en bloc lymphadenectomy with lower neurovascular injury risk (22, 23). Notably, concerns have been raised regarding the accessibility of right recurrent laryngeal nerve (RLN) lymph nodes during transmediastinal esophagectomy, given the anatomic constraints imposed by the trachea and large vessels. However, with proper cervical exposure and angled thoracoscopic instruments inserted through a protective cervical sleeve, our surgical team was able to achieve adequate visualization and dissection along the right tracheoesophageal groove. The combination of CO_2_ insufflation and dynamic mediastinal retraction facilitates exposure of the right RLN course and its associated nodal basin. Although more technically demanding than right thoracoscopic access, bilateral RLN lymphadenectomy is feasible via the transmediastinal route in experienced hands, and our results showed comparable lymph node yields between groups, supporting its oncologic adequacy. Our study demonstrates that this minimally invasive strategy does not compromise surgical quality, as evidenced by similar lymph node yields and resection margins. Notably, patients in the transmediastinal group resumed oral intake earlier, had shorter drainage durations, and experienced fewer Clavien–Dindo grade ≥III complications, marking a significant step forward in aligning oncologic radicality with enhanced recovery after surgery principles. While the overall incidence of anastomotic leakage was relatively low in both groups, the transmediastinal approach demonstrated a favorable profile regarding severity and clinical management. Cervical anastomotic leaks, which occurred in both groups, were more readily managed conservatively in the transmediastinal group, without necessitating reoperation. This may be attributable to earlier detection, less severe clinical manifestations, and the avoidance of intrathoracic contamination. In contrast, one patient in the McKeown group required surgical intervention for leak-related complications. These findings suggest that despite comparable leak rates, the clinical course and management of anastomotic leaks may differ depending on the surgical approach, further supporting the perioperative advantages of the transmediastinal technique.

From an academic perspective, this work contributes to the evolving landscape of esophageal cancer surgery by highlighting a feasible, reproducible, and clinically beneficial technique that directly addresses unmet needs in perioperative safety and patient-centered outcomes. The implications are particularly relevant for patients with impaired cardiopulmonary reserve, advanced age, or previous thoracic interventions, who may otherwise be deemed suboptimal candidates for conventional transthoracic esophagectomy (21, 24). By avoiding thoracic entry and limiting systemic inflammatory response, transmediastinal esophagectomy may reduce postoperative ICU stay and hospital costs, as suggested in previous preliminary series (22). Furthermore, the anatomic clarity afforded by transmediastinal CO_2_ insufflation offers an educational advantage in training programs and has the potential to standardize difficult dissection steps, such as left recurrent laryngeal nerve node removal, across surgical teams with varying experience levels (25).

Nevertheless, the present study is not without limitations. As a retrospective analysis conducted in a single high-volume center, there remains the possibility of selection bias, despite matched baseline characteristics between groups. Surgeons in this study were highly experienced in both techniques, which may limit the generalizability of results to lower-volume institutions or early-phase adoption settings. Furthermore, this analysis focuses on short-term outcomes; the long-term oncological durability of transmediastinal esophagectomy, including overall survival, distant metastasis rates, and anastomotic durability, remains to be clarified through longer follow-up. While the EORTC QLQ-C30 questionnaire was used to assess quality of life at 3 months postoperatively, longer-term functional assessments such as swallowing performance, nutritional status, and chronic pain scores were not included but are of paramount importance to patients and clinicians alike. Furthermore, this study exclusively included patients who underwent upfront surgery without neoadjuvant therapy. This design choice was intended to minimize treatment-related heterogeneity and ensure a uniform baseline for comparing surgical outcomes. However, it also limits the generalizability of our findings to patients who receive neoadjuvant chemoradiotherapy, who constitute a growing proportion of esophageal cancer cases in many centers. Future studies should explore whether the perioperative advantages of transmediastinal esophagectomy persist in the context of pretreated, more advanced disease. Alternative study designs, particularly prospective multicenter randomized controlled trials, are warranted to confirm these findings under higher levels of clinical evidence. Such studies should incorporate stratification by tumor stage, location, and comorbid conditions, and include comprehensive endpoints encompassing immunologic recovery, cost-effectiveness, and long-term survival. In addition, integrating intraoperative adjuncts—such as real-time neuromonitoring, 3D navigation systems, or fluorescence-guided lymphadenectomy—may further enhance the precision and safety of the transmediastinal approach. The potential for robotic assistance also merits exploration, as it may provide better articulation and stability during deep mediastinal dissection, particularly in challenging anatomies such as high BMI patients or those with prior neck surgery. Another promising direction involves applying transmediastinal esophagectomy principles to hybrid or tailor-made surgical plans based on preoperative imaging and physiological risk profiling. Finally, we acknowledge that this study did not include patients with upper thoracic esophageal squamous cell carcinoma. This decision was based on the distinct anatomical challenges and surgical considerations associated with upper thoracic tumors, which often require more extensive cervical and upper mediastinal dissection and are associated with higher risk of recurrent laryngeal nerve injury. Moreover, the incidence of upper thoracic ESCC is relatively low in our center’s surgical cohort during the study period, making it difficult to achieve a sufficiently powered subgroup analysis. Future studies should aim to evaluate whether the perioperative benefits of the transmediastinal approach are similarly applicable to upper thoracic esophageal cancer, potentially through multicenter collaboration and targeted anatomical studies.

Beyond its role in esophageal surgery, transmediastinal esophagectomy exemplifies a broader conceptual shift toward anatomical corridor–based minimally invasive surgery. By exploiting natural passages and avoiding entry into high-risk anatomical compartments, similar strategies may be translatable to the surgical management of mediastinal tumors, thyroid carcinoma with retrosternal extension, or even anterior spine surgeries. As surgical oncology increasingly embraces function-preserving and immune-sparing techniques, the transmediastinal esophagectomy model offers an ideal platform for integrating surgical precision with systemic therapy (22). Future studies should also investigate how transmediastinal esophagectomy affects the perioperative immunological milieu, tumor microenvironment remodeling, and circulating tumor cell clearance—areas where surgical approach may synergize with adjuvant immunotherapies. Approaches such as transmediastinal esophagectomy that align technical innovation with patient-centered outcomes will likely play an increasingly pivotal role.

Additionally, the applicability of thoraco-laparoscopic transmediastinal esophagectomy in patients who have received neoadjuvant chemoradiotherapy (nCRT) warrants careful consideration. While our study exclusively enrolled patients undergoing upfront surgery to ensure homogeneity of baseline conditions, an increasing number of esophageal cancer patients worldwide now receive nCRT as standard preoperative care. This paradigm shift introduces new challenges, including radiation-induced tissue edema, fibrosis, and anatomical distortion, which may complicate transcervical and transhiatal dissection. However, early exploratory studies have suggested that transmediastinal approaches may still be feasible in selected post-nCRT cases, particularly when guided by meticulous preoperative imaging and intraoperative navigation technologies. Future investigations should focus on defining safety thresholds, technical modifications, and patient selection criteria for extending transmediastinal esophagectomy to this growing clinical subset.

In conclusion, thoraco-laparoscopic transmediastinal esophagectomy represents a promising minimally invasive alternative to the traditional McKeown approach for resectable middle and lower thoracic esophageal squamous cell carcinoma. It offers significant advantages in terms of operative efficiency, reduced complication rates, and enhanced postoperative recovery, without compromising short-term oncologic efficacy. While further validation through prospective multicenter studies is needed, our findings support the broader adoption of this technique, particularly in patients with high surgical risk or limited cardiopulmonary reserve.

## Data Availability

The original contributions presented in the study are included in the article/supplementary material. Further inquiries can be directed to the corresponding author/s.
